# Round the World

**Published:** 1916-02-05

**Authors:** 


### ROUND THE WORLD.

[The Editor invites Early Information and Contributions Marked "Fellow-Workers."]

### Fellow-Workers and the Roll of Honour.

Lieutenant-Colonel H. B. Yates, of the Canadian Army Medical Corps, who has died on service, was gazetted lieutenant-colonel in May of last year. He came from Montreal, and two of his sons are serving with the Canadian Contingent.

Lieutenant William Duncan Murray, M.D., who last year was President of the South-West London Medical Society, passed away at his residence at 84 West Side, Clapham Common, on January 23. Dr. Murray was only forty-seven years of age. He obtained his medical training at Glasgow, and, having accepted a lieutenant's commission in the Royal Army Medical Corps, was stationed at Colchester, where he was attached to the Colchester Medical Board. It is only a few months ago that his medical friends in South-West London presented him with a valuable clock. He leaves a widow and two sons, one of whom is at the Front.

Lieutenant A. N. Garrod, R.A.M.C., 100th Field Ambulance, who was killed by a shell in France on January 26, was the eldest son of Dr. A. E. Garrod, well known as physician to St. Bartholomew's and now colonel in the Army Medical Service, and grandson of Sir Alfred Garrod. Lieutenant Garrod received his medical training at Cambridge and at St. Bartholomew's Hospital, obtaining the conjoint qualification M.R.C.S., L.R.C.P. in 1914. Before receiving his commission in July last year he was house surgeon at St. Bartholomew's.

Captain H. H. B. Cunningham, R.A.M.C. (T.F.), attached West Lancashire Divisional Engineers (T.F.), whose name appears among the list of those who have been wounded while on service with the Mediterranean Expeditionary Force, received his medical training at St. Mary's Hospital, London, and at Dublin and Brussels. He obtained the conjoint qualification M.R.C.S., L.R.C.P. in 1903, and became F.R.C.S.I. in 1905. He also held the M.D. degree of Brussels. He was examiner in ophthalmology and otology to the Royal College of Surgeons, Ireland, and held the appointment of ophthalmological surgeon to the Ulster Hospital, Belfast, and was assistant surgeon at the Belfast Ophthalmological Hospital. Prior to his appointment he had been a house surgeon at St. Mary's Hospital.

Captain C. L. Spackman, M.B., R.A.M.C., who has been wounded while on service in the Persian Gulf, received his medical training at Birmingham University, obtaining his medical and surgical degrees in 1912. Subsequently he was house surgeon at the Birmingham General Hospital.

Lieutenant T. E. Hammond, F.R.C.S., R.A.M.C., who is reported wounded, received his medical training at St. Bartholomew's Hospital, obtaining his Fellowship of the Royal College in 1913. He formerly held resident appointments at the West London Hospital.

